# Quantitative Assessment of Sensory Integration and Balance in Children with Autism Spectrum Disorders: Cross-Sectional Study

**DOI:** 10.3390/children9030353

**Published:** 2022-03-03

**Authors:** Mohamed A. Abdel Ghafar, Osama R. Abdelraouf, Abdelgalil A. Abdelgalil, Mohamed K. Seyam, Rafik E. Radwan, Amira E. El-Bagalaty

**Affiliations:** 1 Physical Therapy Program, Batterjee Medical College, Jeddah 21442, Saudi Arabia; 2 Department of Biomechanics, Faculty of Physical Therapy, Cairo University, Giza 12613, Egypt; osamaibrahim2006@gmail.com (O.R.A.); rafik.radwan@gmail.com (R.E.R.); 3 Department of Physical Therapy, Faculty of Applied Medical Sciences, Umm Al-Qura University, Mecca 24382, Saudi Arabia; aaashaaban@uqu.edu.sa; 4 Department of Physical Therapy and Health Rehabilitation, College of Applied Medical Sciences, Majmaah University, Al-Majmaah 11952, Saudi Arabia; m.seyam@mu.edu.sa; 5 Department of Physical Therapy for Pediatric, Faculty of Physical Therapy, Cairo University, Giza 12613, Egypt; seiffaheem@gmail.com

**Keywords:** autism spectrum disorder, sensory integration, balance, Biodex balance test

## Abstract

Postural stability is dependent on the interpretation of external inputs acquired by sensory information processes, such as visual, vestibular, and proprioceptive systems, in order to accomplish neuromuscular control, balance maintenance, and appropriate motor response. A defect in any of these systems, or in the integration of information given by these systems, might threaten their capacity to maintain balance. Therefore, the purpose of this study was to investigate the sensory integration and balance using the Biodex balance system (BBS) in children with autism spectrum disorder (ASD) during the static posture. Seventy-four children from both sexes, 38 with ASD matched with 36 typically developed (TD) children as a control group, were included in the study. Using the Biodex balance system, the postural sway was evaluated through the modified Clinical Test of Sensory Integration and Balance (m-CTSIB) during quiet standing. In this test, four different situations were considered from standing position: eyes open/firm surface, eyes closed/firm surface, eyes open/foam surface, and eyes closed/foam surface. ASD children showed a significant increase in postural sway under all tested conditions when compared to the TD children group, especially for the conditions in which visual and somatosensory inputs were disrupted (*p*-value < 0.05). These results provide evidence that postural stability decreased in ASD children. Under static postural challenges, the current study’s findings imply that children diagnosed with ASD have postural control deficiencies, especially for the conditions in which visual and somatosensory input was disrupted. Further research must be conducted to find the best balance training program for ASD cases using the Biodex balance system and considering its impact on motor skills.

## 1. Introduction

Autism spectrum disorders (ASDs) are a collection of persistent neurodevelopmental disorders characterized by difficulties in social interactions, communication, and repetitive, stereotyped, and restricting behaviors [1]. Its associated characteristics include a diagnosis of intellectual disability, unequal deficits in cognitive skill development, behavioral symptoms, altered reactions to sensory inputs, eating and mood problems, and a variety of non–specific neurological symptoms [2]. Movement problems can occur throughout infancy and are one of the early indications of autism. Furthermore, movement impairments are the most-often-observed nonverbal deficits in autistic children [3].

Because the etiology of ASD is unknown, the particular pattern and source of motor impairments in this group are also unknown. It has been proposed that ASD children may have some impairment in the neural circuits involved in postural stability [4]. The lack of the gamma-aminobutyric acid (GABA) developmental switch in the perinatal stage is a neurodevelopmental theory concerning the mechanism of ASD. Under these situations, proper signals from the limbic system to the cortex are not sent [5]. Behavioral studies have revealed that changes in the structure and functionality of various brain areas, including the cerebellum and the basal ganglia have been detected and linked to problems of posture control. The cerebellum, the key area contributing to sensory integration, was found to have developmental hypoplasia in children with ASD. This may impair motor functioning directly or indirectly through its interactions with the brain stem, thalamus, and hypothalamus [6,7]. Several interrelated structures in the brain play a role in sensory integration and movement execution. Postural control and gait studies in ASD have led to the idea that the abnormalities in postural control may be attributable to a failure in sensory information integration happening in the cerebellum [8].

Postural stability is described as the capacity to maintain an upright posture by keeping the body’s center of gravity over its base of support with little swaying or maximal steadiness, and it is considered a fundamental skill required for normal motor development [9]. Postural stabilization is a complex process that is dependent on sensory information mechanisms, such as the visual, vestibular, and proprioceptive systems, which are responsible for bringing information to the somatosensory cortex, where it is integrated to achieve neuromuscular control, equilibrium maintenance, and appropriate motor response [10]. A deficiency in any of these systems, or in the integration of information given by these systems, may impair the capacity to maintain equilibrium [11]. 

ASD is well-known for having social interaction impairments. According to the findings of a recent study, ASD’s poor postural control and weaker visual strategies may be related to a lack of interest in social cognition, which produces a delay in the development of the cortical regions, and thus has a negative effect on their postural control [12]. When sensory input information is blocked or deleted, children with ASD have a diminished capacity to maintain postural stability during static and dynamic postures, including greater postural sway [13]. Impaired postural control may have a significant influence on the development of perceptual-motor skills and social functioning in children with ASD [14]. 

Minshew et al. [4], stated that both children and adults with ASD show reduced postural stability when compared to persons with typical development (TD) in situations where one or more sensory inputs were removed or adjusted. Reed and McCarthy [15] investigated postural control with and without an extra auditory task during a visual task. They discovered that when children with ASD had to integrate both visual and audio stimuli, they were more unstable than controls.

An impaired postural control system can limit the development of other motor skills, limit the capacity to acquire mobility and manipulatory skills, and have a substantial impact on quality of life. Motor deficiencies in the ASD population have received little attention and been dismissed as a minor or co-occurring condition [16].

A review of the literature revealed that multiple posturography approaches have been utilized, in addition to varied postural control variables. Each measuring technique can investigate a different aspect of postural control. Furthermore, because each sway parameter accounts for a distinct aspect of postural control, interpretation of results should be dependent on the precise characterization of each variable and the instrument used [17]. According to Dawson et al. [18], there is limited construct validity between the foursquare step test, timed up and go test, Biodex SD assessments of stability limits, and the modified Clinical Test of Sensory Integration and Balance. They proposed that professionals should employ more than one tool to examine different elements of a patient’s balance deficiencies to better plan therapy and intervention. 

To the best of our knowledge, no previous study has used the Biodex balance system (BBS) to investigate postural stability in children with ASD. Therefore, the purpose of this study was twofold; first, objectively assess sensory integration and balance in children with ASD using BBS during quiet standing as compared to TD controls. Second, investigate the effect of age and IQ of the participants on postural stability in children with ASD. 

## 2. Materials and Methods

### 2.1. Study Design

This is an observational cross-sectional study. The study was authorized by the institutional Ethics Committee of Batterjee Medical College (RES-2020-0022) in compliance with the ethical standards of the Declaration of Helsinki. 

### 2.2. Participants

Seventy-four children, 38 with ASD (25 boys and 13 girls) and 36 TD children (21 boys and 15 girls) as a control group, matched at the group level in age, sex, and body mass index (BMI), participated in this study. The age of the study group ranged from 6 to 14 years. The study took place in the period between June 2020 and October 2021.

**Figure 1:**
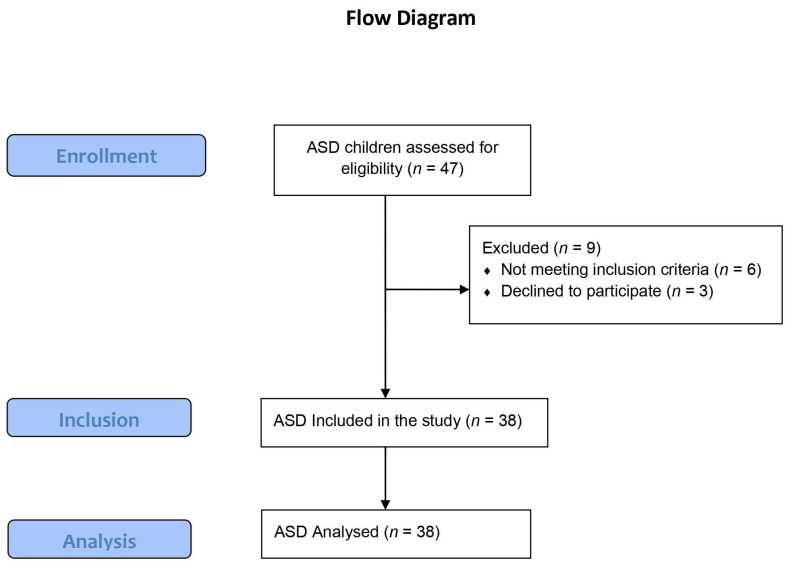
The flow diagram of the ASD children through the stages of the study.

Children with ASD were recruited from the local autism center based on the following eligibility criteria: have an IQ ≥ 70 according to Stanford–Binet Intelligence Scales and be able to understand and follow instructions. A diagnosis of level one (mild) ASD was established using the Diagnostic and Statistical Manual of Mental Disorders, fifth edition (DSM-5), and was performed by a psychiatrist specialized in autism. Children were excluded if they were using medicines known to impair motor function at the time of testing, such as antipsychotics, stimulants, or anticonvulsants, had a history of head injury, genetic disorders or congenital abnormalities, gross sensory deficits such as blindness or hearing loss, use of assistive devices, diagnosis of psychiatric or neurological disorders, recent fractures or musculoskeletal disorders [19]. To investigate the effect of age and IQ on postural stability, the participants were further subdivided into two groups: 6–9 years and 10–14 years based on age and 70–89 scores and ≥90 based on IQ score. Figure 1 shows a flow diagram of the subject’s selection. Written informed consent from the child’s legal guardian was obtained after the experimental procedure was explained to them. Demographic characteristics data of the participants are shown in Table 1. The sample size was calculated to be 34 participants using G-power 3.1 based on α = 0.05, power = 0.80, and an effect size = 0.50.

### 2.3. Procedures

#### 2.3.1. Evaluation Procedures

##### Assessment of IQ

The Stanford–Binet Intelligence Scales, 5th edition (SB5), was used to test IQ. It is one of the most common intelligence measures, and it is especially well-represented in the intellectual and developmental disorders sector. The SB5 assesses overall intellectual capacities and allows for the reporting of full-scale, abbreviated, nonverbal, and verbal IQ scores [20]. This measure also includes five categories that are closely connected to the Cattell–Horn–Carroll hierarchical model of cognitive talents. Fluid reasoning, knowledge, quantitative reasoning, visual–spatial processing, and working memory are among these elements [21].

##### Balance Assessment

Biodex balance system SD (Balance SD, 115 VAC Biodex Medical Systems, Inc. 20 Ramsey Road, Shirley, NY, USA) was used in this study to assess static balance. BBS is proven to be a reliable and valid method for objectively assessing postural stability [18,22].

**Figure 2:**
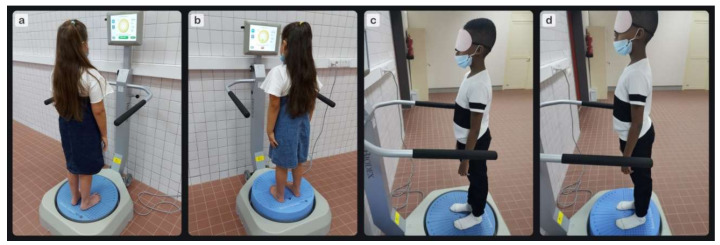
This is a figure for the testing procedures. (**a**) Eyes open/firm surface, (**b**) eyes open/foam, (**c**) eyes closed/firm surface, (**d**) eyes closed/foam.

Modified Clinical Test of Sensory Integration and Balance (m-CTSIB) was used. The Biodex balance test procedure is intended to provide a general assessment of an individual’s capacity to integrate different senses in terms of balance and to adjust when one or more of these senses is impaired. Before performing the test, each child had three familiarization trials that were not counted in measurement. In this test, four different situations were considered from the standing position at the center of the balance system platform with their feet shoulder-width apart: eyes open/firm surface, eyes closed/firm surface, eyes open/foam surface, and eyes closed/foam surface. During the last two conditions, a foam pad with the same markings as the firm surface was placed on the platform. Each participant was told to stand as long as they could for the whole 20 s for each of the four testing situations, with a 10 s rest period in between [18]. It was challenging for children with ASD to stay attentive and motionless for an extended amount of time. As a result, a short test duration was utilized [23]. The order of testing was randomized to avoid the effect of learning. The sway index was recorded for each condition of each trial, and the average of each condition was calculated. The higher the sway index, the more unsteady the person was during the test. Testing procedures are shown in Figure 2.

#### 2.3.2. Statistical Analysis

For collected data analysis, the Statistical Package for Social Sciences (SPSS) for Windows version 20.0 was used (SPSS, Armonk, NY, USA, IBM Corp.). Numerical data were expressed as mean and standard deviations. The normal distribution and homogeneity of the data were checked prior to conduction of data analysis using Shapiro–Wilk and Levene’s tests (*p* > 0.05). Accordingly, parametric tests were valid for statistical analysis in this study. Only, a chi-square test was used to determine the difference in gender, while unpaired *t*-test was used to determine the difference in age, height, weight, and BMI between both groups (*p* > 0.05). Multivariate analysis of variance (MANOVA) was used to compare the stability indices of all tested conditions: eyes open/firm surface, eyes closed/firm surface, eyes open/foam surface, and eyes closed/foam surface in both groups based on age and IQ score. If the F-ratio is significant, Tukey HSD post hoc test was used for subsequent multiple pairwise comparisons. The significance level of a *p*-value of ≤0.05 was considered statistically significant using 95 percent confidence intervals.

## 3. Results

Seventy-four children from both sexes (38 with ASD and 36 TD children) with an age range from 6 to 14 years participated in this study. Table 1 showed no significant difference in age, height, weight, BMI, IQ, and sex between both groups (*p* > 0.05), except for IQ level (*p* < 0.05). Between-group comparisons revealed a significant difference in the postural sway index between the ASD group and TD group in all the tested conditions: eyes open/firm surface, eyes closed/firm surface, eyes open/foam surface, and eyes closed/foam surface *p*-value < 0.05. Within-group comparisons based on age showed that the ASD children aged between 10 and 14 years had significantly lower postural sway scores during eyes open/firm surface and eyes open/foam surface testing conditions (*p* > 0.05), with a non-significant difference between eyes closed/firm surface and eyes closed/foam surface testing scores (*p* < 0.05) compared with ASD children aged between 6–9 years, as shown in Table 2. On the other hand, within-group comparisons based on IQ level showed that ASD children with a score of ≥90 had a lower postural sway score in all testing conditions as compared with those who had an IQ score between 70 and 89, as shown in Table 3. 

## 4. Discussion

The purpose of this study was to investigate the sensory integration and balance in children with ASD during quiet standing in different testing conditions (eyes open/firm surface, eyes closed/firm surface, eyes open/foam surface, and eyes closed/foam surface) by using Biodex m-CTSIB, as well as investigating the effect of age and IQ of children with ASD on postural stability. To the authors’ knowledge, this is the first study to use Biodex as an objective assessment tool for balance and sensory integration in children with ASD. The Biodex m-CTSIB test measures a patient’s sway index, which is a method of estimating the mean absolute deviation of the patient’s trunk average position during a test. The greater the sway index, the more unstable the person was during the test. The results of the current study showed that children with ASD have exacerbated postural sway, compared with TD in all tested conditions, especially for the conditions in which visual and somatosensory inputs were disrupted according to m-CTSIB. These results confirmed that ASD children tended to rely on visual cues the most to prevent sway and maintain balance. Regardless of whether somatosensory input was altered, children with ASD experienced a much higher increase in sway when their vision was blocked compared to controls. The pattern of sway responses we found is more compatible with a deficiency in sensory integration than a defect in a specific afferent pathway [9]. 

The results of this study can be explained by the statement of Stins and Emck [24] that the body is in near postural balance during upright standing, but external and internal disturbances demand postural modifications to prevent loss of stability. This process entails integrating sensory inputs to appropriately identify postural orientation, and then executing adequate motor instructions to restore postural stability. Balance control is not solely reflex-driven; higher regions such as the motor cortex, basal ganglia, cerebellum, vestibular cortex, and brainstem are also involved. Several studies on autism discovered that not only is postural control disturbed, but that these postural disturbances are predictive of ASD.

The findings of this study were in parallel with the previous findings of Molloy et al. [9], who reported that ASD children had larger sway areas, which increased task difficulty, and Graham et al. [25], who concluded that children with ASD have worse postural stability and more postural sway compared to the normal children. Furthermore, the results of Fournier et al. [1] indicated that ASD children display postural instability during quiet standing even when no sensory manipulations are used. Additionally, Adamovic et al. [26] analyzed the ability to maintain postural balance in children with autism in comparison to typically developed children, finding that children with autism manifest poorer postural balance than their peers, compared to the typical population.

Additionally, the results of this study came in agreement of Gouleme et al. [12] who concluded that ASD children showed a lower postural control and deficiencies in the visual strategy used to examine emotional faces when compared to TD peers. Furthermore, as children with ASD explored happy and sad emotional expressions, their postural sway increased. Moseley and Pulvermüller [27] recently reported that ASD is characterized by a wide spectrum of modest motor control impairments, including postural instability, which may eventually impede normal cognitive and social development.

Moreover, the results of this study came in partial agreement with Stins et al. [28] who compared a group of children with mild ASD to a group of matched normal controls on several postural activities while standing on a Wii Balance Board. The authors found that closing the eyelids had a more destabilizing effect on the ASD group than on the controls, which means that the children with ASD rely more on their vision to maintain their balance.

Many previous studies showed that sensory information processing and sensory regulation of balance function suboptimally in ASD. The meta-analysis of Lim et al. [29] highlighted how sensory processing deficits negatively influence postural balance in ASD. ASD displayed increased postural sway in response to practically all types of visual and somatosensory manipulations, and they hypothesized that this group’s ability to integrate information from diverse sensory pathways into an adequate motor response was hindered.

On the other hand, regarding the first testing condition, the results of this study came in disagreement with Stins et al. [28], who discovered that, at baseline, eyes open and firm surface, when compared to controls, children with mild autism did not vary in their sway. Methodological differences may have contributed to the findings in this study such as using a different balance assessment tool, and the low sample size in the study by Stins et al., in which only nine children with ASD were included.

Furthermore, Doumas et al. [13] reported that, during quiet standing, both visual and proprioceptive information became less reliable. A group of individuals with ASD and a group of controls were studied for postural sway. It was discovered that discrepancies in postural instability across groups increased in proportion to the inaccuracy of sensory input. This shows that people with ASD are hyper-reactive to sensory disruptions.

Moreover, empirical evidence suggested that increased postural sway in ASD children was caused in part by the impaired integration of vestibular, somatosensory, and visual signals [4]. The study of Cordeiro et al. [30] concluded that autistic children did not have difficulty executing the pediatric balance scale and sensory organization test and received scores near to the maximum. According to the examination using these tests, ASD children did not have postural balance problems.

The results of this study regarding the effect of IQ on postural stability showed that a lower IQ score was associated with poor postural stability in children with ASD. These results were supported by Kaur et al. [31] who found that children with intellectual impairment and ASD, aged 4 to 12 years, scored considerably lower in balance, posture, and body coordination, as well as gross motor skills. Moreover, these findings aligned with Ramos-Sánchez et al. [32], who concluded that balance is moderately influenced by performance IQ in ASD children.

Our results regarding the effect of age on postural stability revealed that postural stability improved in some aspects with increased age. These results came in agreement with Yumeng et al. [33], who concluded that certain age-related alterations in postural control were identified in children with ASD. The U14 group had better mediolateral postural stability than the U8 group. These findings may imply that ASD children may gradually improve postural stability but only show meaningful changes over a lengthy period.

The current study has some limitations. First, the evaluation of the vestibular system was not included because this study adopted the modified CTSIB assessment protocol. Secondly, the severity of autism and its effect on postural control were not studied. Moreover, as the design of this research was an observational cross-section, future investigations about the effects of the BBS as a training tool in ASD children are recommended.

## 5. Conclusions

The current study findings suggest that there was a significant impairment in postural control in children diagnosed with ASD when compared to TD controls under static postural challenges. When compared to TD children, postural control testing revealed that a failure in the integration of sensory input from the visual and somatosensory afferent systems leads to postural instability in children with ASD. Furthermore, lower stability was related to low IQ scores and, in some aspects, younger ages. By better characterizing the postural impairment associated with ASD using more objective tools such as BBS, which can digitally examine the interplay of the sensory systems responsible for postural control and detect even the smallest body sway, the early intervention with balance training may help to prevent the emergence of deficits in other motor abilities.

## Figures and Tables

**Table 1:** The demographic characteristics of ASD and TD children.

Groups	ASD Children, (*n* = 38)	TD Children, (*n* = 36)	*p*-Value
	Mean ± SD	Mean ± SD	
Age (years)	9.57 ± 2.08	10.84 ± 2.91	0.911
Height (m)	1.39 ± 9.72	1.31 ± 5.104	0.648
Weight (kg)	41.68 ± 9.13	37.45 ± 5.68	0.895
BMI	20.56 ± 6.41	19.89 ± 3.05	0.917
IQ	95 ± 11	115 ± 9	0.001
Sex (girls/boys)	13/25	15/21	0.827

Data are illustrated as mean ± standard deviation, ASD (autism spectrum disorder), TD (typical development), IQ (intelligence quotient), *p* value > 0.05 means non-significant.

**Table 2:** Comparison of postural stability testing conditions of ASD and TD children based on age.

Variables		Age		*p*-Value
		6–9 Year	10–14 Year	
		Mean ± SD	Mean ± SD	
Eyes open/firm surface	ASD children	5.73 ± 1.34	3.26 ± 1.15	0.017 *
	TD children	0.71 ±0.07	0.59 ± 0.03	0.022 *
	*p*-value	0.001 *	0.002 *	
Eyes closed/firm surface	ASD children	5.24 ± 1.71	4.47 ± 1.72	0.526
	TD children	1.69 ± 0.09	1.07 ± 0.08	0.020 *
	*p*-value	0.001 *	0.007 *	
Eyes open/foam surface	ASD children	5.06 ± 2.21	3.16 ± 0.87	0.015 *
	TD children	1.34 ± 0.02	0.97 ± 0.3	0.024 *
	*p*-value	0.001 *	0.005 *	
Eyes closed/foam surface	ASD children	7.21 ± 1.68	6.78 ± 2.11	0.670
	TD children	2.02 ± 0.26	1.45 ± 0.13	0.018 *
	*p*-value	0.001 *	0.001 *	
Overall sway index score	ASD children	6.58 ± 0.62	4.25 ± 1.15	0.021 *
	TD children	1.50 ± 0.04	1.03 ± 0.07	0.017 *
	*p*-value	0.001 *	0.007 *	

Data are illustrated as mean ± standard deviation, ASD (autism spectrum disorder), TD (typical development), * significant difference (*p* < 0.05).

**Table 3:** Comparison of postural stability testing conditions of ASD and TD children based on IQ score.

Variables		IQ Score		*p*-Value
		70–89	≥90	
		Mean ± SD	Mean ± SD	
Eyes open/firm surface	ASD children	6.02 ± 1.05	3.89 ± 1.78	0.020 *
	TD children	0.64 ±0.14	0.62 ± 0.06	0.802
	*p*-value	0.001 *	0.003 *	
Eyes closed/firm surface	ASD children	5.87 ± 1.08	3.1 ± 0.99	0.019 *
	TD children	1.49 ± 0.28	1.42 ± 0.43	0.801
	*p*-value	0.001 *	0.006 *	
Eyes open/foam surface	ASD children	5.21 ± 2.06	3.27 ± 0.98	0.021 *
	TD children	1.25 ± 0.11	1.29 ± 0.07	0.789
	*p*-value	0.002 *	0.018 *	
Eyes closed/foam surface	ASD children	6.92 ± 1.97	4.62 ± 0.73	0.021 *
	TD children	1.83 ± 0.51	2.00 ± 0.28	0.425
	*p*-value	0.001 *	0.019 *	
Overall sway index score	ASD children	6.82 ± 0.38	3.45 ± 0.35	0.020 *
	TD children	1.45 ± 0.09	1.06 ± 0.1	0.022 *
	*p*-value	0.001 *	0.006 *	

Data are illustrated as mean ± standard deviation, ASD (autism spectrum disorders, TD (typical development), * significant difference (*p* < 0.05).

## Data Availability

The materials that support this manuscript are available from the corresponding author upon reasonable request.
